# Editorial: Innovative Approaches in Diagnosis of Emerging/Re-emerging Infectious Diseases

**DOI:** 10.3389/fmicb.2020.619498

**Published:** 2020-12-03

**Authors:** Aleksandra Barac, Mario Poljak, David S. Y. Ong

**Affiliations:** ^1^Clinic for Infectious and Tropical Diseases, Clinical Center of Serbia, Belgrade, Serbia; ^2^Faculty of Medicine, University of Belgrade, Belgrade, Serbia; ^3^Institute of Microbiology and Immunology, Faculty of Medicine, University of Ljubljana, Ljubljana, Slovenia; ^4^Department of Medical Microbiology and Infection Control, Franciscus Gasthuis & Vlietland, Rotterdam, Netherlands; ^5^Department of Epidemiology, Julius Center for Health Sciences and Primary Care, University Medical Center Utrecht, Utrecht University, Utrecht, Netherlands

**Keywords:** infectious diseases, emerging infections, outbreak, pandemic infections, diagnosis, treatment

Infectious diseases can spread rapidly, causing enormous losses of health, human lives, as well as large costs to the society. Despite recent significant advances in diagnostics of infectious diseases, the frequent and affordable worldwide travel which was common before coronavirus disease 2019 (COVID-19) pandemic and increased global interdependence have added layers of complexity to recognize and timely manage several infectious diseases, including both traditional as well as emerging/re-emerging diseases (Castelli and Sulis, 2017). HIV/AIDS, multidrug-resistant tuberculosis (TB), invasive fungal infections in transplant recipients, *Clostridioides difficile* enterocolitis, malaria, measles, severe acute respiratory syndrome (SARS), Middle East respiratory syndrome (MERS), pandemic avian and H1N1 influenza, Ebola and Zika are only a few of many examples of infectious diseases which emerged in the last decades causing significant morbidity and mortality (Rechel et al., 2013). In addition, new infectious diseases with epidemic and pandemic potential emerge periodically. In extreme cases they may cause devastating pandemics such as COVID-19, whereas in other cases they result in self-limited infections or local/regional epidemics of smaller scale (Morens and Fauci, 2020). Established traditional infectious diseases may also re-emerge, for example by extending geographically or when pathogens become more transmissible, more pathogenic or resistant to standard antimicrobial therapy. Disease emergence reflects dynamic balances and imbalances, within complex globally distributed ecosystems comprising humans, animals, pathogens, and the environment. To successfully control emerging/re-emerging infectious diseases, the best strategy is to understand these variables and to halt their spread at an early stage. For this purpose, we rely on early diagnosis of disease by rapid and reliable detection of disease-causing agents. This is particularly true for newly emerging infectious disease (like SARS-CoV-2 and MERS), where we find ourselves in a race to develop and validate new diagnostic tools and approaches during an ongoing outbreak.

This Research Topic entitled “Innovative Approaches in Diagnosis of Emerging/re-emerging Infectious Diseases” includes a total of 18 manuscripts, ranging from original research to reviews within all fields of microbiology: bacteriology, virology, mycology and parasitology; all with a particular focus on diagnostics. It covers research on highly-prevalent infectious diseases such as tuberculosis and HIV, but also on emerging tropical and fungal infectious diseases.

Several new diagnostic approaches for diagnosis of TB have been studied in the last decade. This Research Topic includes four articles on TB (Zhou et al.; Jiao et al.; Dusthackeer et al.; Yong et al.). The study by Zhou et al. showed that the Ku protein is important in maintaining the genome's integrity of mycobacteria and that the ku gene was a highly specific biomarker for *Mycobacterium tuberculosis* (MTB) complex and non-tuberculosis *Mycobacteria*, which makes differentiation of *Mycobacteria* to closely related bacteria possible by a simple non-sequencing molecular method (Zhou et al.). In the study by Jiao et al. a design and evaluation of novel diagnostic assay was reported, termed multiple cross displacement amplification combined with nanoparticle-based lateral flow biosensor (MCDA), developed to simultaneously detect IS6110 and IS1081 of *Mycobacterium tuberculosis* (MTB) (Jiao et al.). Among clinically diagnosed TB patients, the sensitivity of liquid culture, Cepheid Xpert MTB/RIF and newly developed multiplex MCDA assay was 42, 50% and 88%, respectively. Among culture positive samples, the sensitivity of Xpert MTB/RIF and multiplex MCDA assay was 86 and 98%, respectively. Among culture negative samples, the sensitivity of Xpert MTB/RIF and multiplex MCDA assay was 23.2 and 81.2%, respectively. The specificity was 100% for Xpert MTB/RIF and 98.3% for multiplex MCDA. The results showed that the multiplex MCDA assay is a rapid, sensitive and easy to use tool for early diagnosis of TB (Jiao et al.). In the study of Dusthackeer et al., authors reported the presence of non-replicating bacteria in the sputum of suspected TB cases that show differential growth response to resuscitation-promoting factors (RPFs) treatment. Crude and recombinant RPF treatment improved sensitivity and reduced time to detect bacilli in the sputum samples of smear-positive/culture-negative or smear-negative/culture-negative cases. These findings have implications for improving the bacteriological diagnostic modalities currently in use for diagnosis of TB in endemic countries. New diagnostic approaches reported in this Research Topic may overcome the limitations observed with the currently available TB diagnostic tools (Acharya et al., 2020). This Research Topic also includes a review by Yong et al. on recent advances on TB biomarkers, particularly host biomarkers that have the great potential to improve diagnosis and differentiate between active and latent TB as well as on their use in disease prognosis and treatment monitoring (Yong et al.).

Another focus in this Research Topic is on novel approaches in diagnosis and treatment of emerging/reemerging tropical diseases (Shrivastava et al.; Mondal et al.; Buitrago and Martín-Gómez). Shrivastava et al. described design and evaluation of a sensitive and specific IgG indirect ELISA for high-throughput screening of Crimean-Congo hemorrhagic fever using viral multispecies recombinant nucleoprotein as an antigen. Results of Mondal et al. study (Mondal et al.) indicate that the relative quantity of serum anti-rK39 antibody is promising predictive and/or prognostic marker for visceral leishmaniasis or disease-related modalities. These could enable control programs to implement more effective measures to eliminate visceral leishmaniasis. More effective measures are also needed for histoplasmosis, which is increasingly reported in non-endemic countries as a result of recreational travels and migratory movements. Timely diagnosis is hampered by the lack of clinical awareness, and the scarcity of laboratory tools able to provide accurate results with a short turn-around time regardless of the immune status of the host and the extent of the disease. Molecular techniques are seen as a suitable alternative for this purpose in low prevalence areas and much needed efforts to standardize such assays and to define their diagnostic yield are in progress. Buitrago and Martín-Gómez reviewed current evidence behind the use of molecular tests in non-endemic areas, and as adjunctive tests for the laboratory diagnosis of histoplasmosis in high endemic areas (Buitrago and Martín-Gómez).

Two articles in this Research Topic evaluated new methods or combination of existing methods for more timely diagnosis of fungal infections (Vaezi et al.; Lo Cascio et al.). Vaezi et al. showed that the microculture assay may be useful for rapid culturing and diagnosis of mucormycosis caused by *Rhizopus arrhizus* directly in blood and tissue samples. Hence, this method may allow more timely administration of an appropriate treatment (Vaezi et al.). Despite that whole-genome sequencing is universally accepted as a reference method for identification of rare emerging pathogens, Lo Cascio et al. showed that proteomics is a promising alternative to laborious and time-consuming molecular methods for identification and evaluation of strain clonality in outbreaks of rare pathogens (Lo Cascio et al.).

Two contributions in this Research Topic reported results of studies in pediatric populations (Carter et al.; Fu et al.). Carter et al. assessed an antibody-in-lymphocyte supernatant assay for the etiological diagnosis of pneumococcal pneumonia in children (Carter et al.) and concluded that this method detected spontaneous secretion of IgG to pneumococcal protein antigens from cultured peripheral blood mononuclear cell. However, when stratified by age groups, this assay showed limited utility as a test to discriminate between pneumococcal and non-pneumococcal pneumonia in children. Fu et al. focused on autoinducer-2 (AI-2), which has a widely accepted role in bacterial intra- and interspecies communication (Fu et al.). This study found that AI-2 levels in a necrotizing enterocolitis mouse model as well as in patients correlate with intestinal flora disorder and different stages of disease. Based on these findings, AI-2 may be a new biomarker for the diagnosis and monitoring of necrotizing enterocolitis.

The possible new clinical applications of molecular methods were shown in two articles in this Research Topic (Sheikh et al.; Weng et al.). Sheikh et al. developed a colorimetric loop-mediated isothermal amplification assay (LAMP) for detection of IS1111a gene of *Coxiella burnetti* in sheep vaginal swabs (Sheikh et al.). LAMP is a more rapid and simpler method without the need for extensive sample preparation in comparison to routine PCR assay, whereas the sensitivity in Sheikh et al. study was comparable between both approaches. Moreover, LAMP could especially be attractive as point-of-care test in resource-limited settings (Ong and Poljak, 2020). Weng et al. showed that next-generation sequencing is a promising tool for rapid diagnosis of nocardiosis and could greatly reduce the turnaround time in comparison to traditional culturing methods, but current high cost and low accessibility of next-generation sequencing still limit its utility in daily practice (Weng et al.).

Despite the great promise of newly developed breakthrough molecular methods, traditional molecular approaches remain indispensable in several clinical situations. Nikolic et al. described challenging diagnostic case of rare Lyme endocarditis diagnosed by traditional PCR. As the manifestations of Lyme endocarditis are non-specific, clinicians should test heart valve samples by PCR in case of endocarditis of unknown origin, especially in Lyme disease endemic areas (Nikolic et al.).

Innovative and practical diagnostic approaches in the field of viral infections were also evaluated in this Research Topic (Breznik et al.; Tuaillon et al.). Breznik et al. detected human papillomaviruses (HPV) in 95% of common warts and showed that type-specific quantitative real-time PCRs could reliably assign the causative HPV type in almost 80% of cases, which provides further evidence that could be used in the selection of the most appropriate targets for future vaccines against HPV-related cutaneous tumors (Breznik et al.). Tuaillon et al. reviewed the current knowledge concerning the use of dried blood spots (DBS) for the diagnosis and therapeutic monitoring of HIV, hepatitis B and hepatitis C. DBS is a valid alternative in settings where there is no possibility for taking venous whole blood specimens or where transport of body fluids is difficult (Tuaillon et al.). Although DBS samples are suitable for testing of the presence of antibodies, antigens or nucleic acids, the lower analytical sensitivity remains one of the major limits of DBS in comparison to traditional blood samples. Therefore, the advantages of decentralized sampling and improved access to testing should be carefully outweighed against the possible limitations.

Finally, this Research Topic also includes articles on respiratory viruses, such as a review by Nelson et al. on current and future use of point-of-care tests (POCTs) for traditional and emerging respiratory viruses (Nelson et al.). During COVID-19 pandemic the use of POCTs has become more important than ever before. This review provides a description of new and innovative diagnostic techniques, ranging from biosensors to novel portable and current lab-based nucleic acid amplification methods with the potential future use in POCTs settings. In addition, Guo et al. designed an easy-to-use clinically predictive tool for assessing 90-days mortality risk of viral pneumonia. The tool can accurately stratify hospitalized patients with viral pneumonia into relevant risk categories and could provide guidance for further clinical decisions.

## Conclusion

The original research and review articles in this Research Topic highlighted the importance and constant need for the development of novel, rapid and more accurate diagnostic tools for diagnosis of emerging/re-emerging infectious diseases. Contributions in this Research Topic described recent innovative approaches and provide hopeful prospects for improved patient care. Hopefully, this Research Topic will also stimulate further research with the ultimate goal of improving diagnosis and outcomes for patients with emerging infectious diseases, which should not be limited to the eminent threat by the current SARS-CoV-2 pandemic.

## Author Contributions

All authors listed have made a substantial, direct and intellectual contribution to the work, and approved it for publication.

## Conflict of Interest

The authors declare that the research was conducted in the absence of any commercial or financial relationships that could be construed as a potential conflict of interest.
